# Staff and service users’ views on a ‘Consent for Contact’ research register within psychosis services: a qualitative study

**DOI:** 10.1186/s12888-014-0377-6

**Published:** 2014-12-24

**Authors:** Constantina Papoulias, Dan Robotham, Gareth Drake, Diana Rose, Til Wykes

**Affiliations:** Department of Psychology, Institute of Psychiatry, King’s College London, London, UK; Department of Clinical, Educational & Health Psychology, University College London, London, UK; Service User Research Enterprise, Institute of Psychiatry, King’s College London, London, UK

**Keywords:** Informed consent, Health records, Research register, Recruitment, Service user involvement, Psychosis

## Abstract

**Background:**

Recruitment to mental health research can be challenging. ‘Consent for Contact’ (C4C) is a novel framework which may expedite recruitment and contribute to equitable access to research. This paper discusses stakeholder perspectives on using a C4C model in services for people with psychosis.

**Method:**

This is a cross sectional study investigating the views of service users and staff using qualitative methods. Eight focus groups were recruited: five with service users (n = 26) and three with clinicians (n = 17). Purposive sampling was applied in order to reflect the local population in terms of ethnicity, experience of psychiatric services and attitudes towards research.

**Results:**

Staff and service users alike associated the principle of ‘consent for contact’ with greater service user autonomy and favourable conditions for research recruitment. Fears around coercion and inappropriate uses of clinical records were common and most marked in service users identifying as having a negative view to research participation. Staff working in inpatient services reported that consenting for future contact might contribute to paranoid ideation. All groups agreed that implementation should highlight safeguards and the opt-in nature of the register.

**Conclusions:**

Staff and service users responded positively to C4C. Clinicians explaining C4C to service users should allay anxieties around coercion, degree of commitment, and use of records. For some service users, researcher access to records is likely to be the most challenging aspect of the consultation.

## Background

Research is essential for the improvement of services and treatments in mental health. However, recruitment to studies can be difficult as access to potential participants frequently depends on complex extended negotiation with care teams. This process is often cumbersome, and may delay research outputs, while also placing extra strain on services and staff. Furthermore, care team mediation can result in ‘gate-keeping’ thereby restricting service user access to research and potentially contributing to biased sampling [1,2]. In the UK such a restriction would be out of step with current policy which emphasises patient autonomy and equitable access to health research [3]^a^. In this context, alternative and more expedient recruitment methods are being sought [4].

The NIHR Biomedical Research Centre at the South London and Maudsley National Health Service Foundation Trust (SLaM) and King’s College London is implementing a novel recruitment method which makes use of Electronic Health Records for the identification of potential participants for studies^b^. Under the Data Protection Act 1998, use of identifiable health records for the purposes of research requires prior individual informed consent. Therefore, the SLaM model involves a ‘Consent for Contact’ register (C4C) which allows researchers with ethically approved studies to access identifiable service user records for the purposes of recruitment. A detailed account of the C4C model and its negotiation of consent in mental health research is published elsewhere [5]. It is hoped that C4C will facilitate recruitment, reduce ‘gate-keeping’ and contribute to increased rates of service user engagement in mental health research, in accordance with NHS policy.

The Clinical Record Interactive Search (CRIS) aggregates and de-identifies the electronic health records in SLaM, thus making them searchable while preserving service user anonymity [6,7]. The SLaM C4C register makes use of CRIS for the selection of potential participants in research studies. This means that the records of service users on the register can be de-anonymised under strict conditions within the NHS firewall, if they match inclusion criteria for specific studies. Implementation of C4C requires that a member of a service user’s clinical team explains the C4C register and asks whether the service user would give generic consent to future contact by researchers. Staff members must clarify that if a service user consents for contact, they *“are willing to be contacted about current and future research projects and are willing for researchers to identify them from their case records”* (to adjudicate if they are potentially suitable for recruitment to their particular study). Each service user’s decision is then entered on a specific section of their electronic health record. The record also includes a “comments box” where service users can specify whether there are parts of their records they do not wish to be contacted about (such as substance misuse, traumatic experiences and so on). Finally, service users who have joined the register are able to withdraw their consent by asking a member of their clinical team to update their record. This system was designed with service user input and collaboration and is governed by an Oversight committee that is chaired by a service user researcher.

Even though the development of C4C was participatory there are still many barriers to the acceptance of a register – these were highlighted following the recent attempts at informing the public about the UK care.data programme [8]. The use of identifiable records for research may pose additional challenges in mental health. There is some evidence that mental health service users may be more reluctant to consent to research uses of their health records than the general population insofar as they may be more likely to perceive these records as potentially stigmatising [9,10]. Furthermore, mental health service users’ attitudes to services may be ambivalent and in some cases, hostile, since such services are, for some service users, associated with past or potential involuntary detainment. Lastly, people who experience paranoid ideation and delusions involving government conspiracies may be more anxious about the possibility of unauthorised access [11].

Therefore we have continued with our participatory approach and investigated service user and clinician perceptions of a C4C register in order to understand and address potential challenges and develop a comprehensive training programme for staff, which provides support for answering service user concerns.

## Method

### Design

This cross sectional study investigated the views of service users and staff using qualitative methods. Eight focus groups were recruited (five service user and three staff) from different types of psychiatric service for people with psychosis. Purposive sampling was applied in order to reflect the local population in terms of ethnicity, experience of psychiatric services and attitudes towards research, with the aim of obtaining a wide range of service user perspectives on C4C. Social workers, community psychiatric nurses (CPNs) and psychologists were targeted for the staff focus groups, since it is these workers who, in their capacity as primary health workers (known as ‘care co-ordinators’), act as a regular point of contact for mental health service users within secondary services in the NHS. Furthermore, researchers usually rely on care co-ordinators to access potential study participants.

### Sample

Three clinician and five service user groups were convened (n = 17 and n = 26 respectively). The clinician groups and three of the service user groups were recruited from community services, an acute inpatient ward and a specialised early intervention service (n = 5,6,6 and n = 7,4,4 respectively). A fourth service user group was composed of black and ethnic minority participants, reflecting service demographics (n = 5). The fifth group included service users who self-identified as having a ‘negative’ attitude to research participation (n = 6), recruited so that the concerns of people likely to withhold consent could be investigated. The ‘research negative’ group was recruited through posters and leaflets in the vicinity of the local hospital and all the other groups through psychosis services.

### Data collection

Focus groups were facilitated by two service user researchers. A topic guide, developed through consultation with a service user reference group, was used to present the C4C register and to prompt questions on the following: how and when to explain C4C to service users, positive and negative aspects of the model, thoughts about improving the model, reasons for withholding consent. Group discussions were audio-recorded, transcribed verbatim and checked for errors by two service user researchers.

All participants were given written information about the study and provided informed consent. The study was approved by the National Research Ethics Service, London - Dulwich (reference: 11/LO/1255).

### Data analysis

NVivo10 was used for thematic analysis of the focus group transcripts – the topic guide supplied the coding frame, while data were also analysed inductively to uncover supplementary themes [12]. Two service user researchers analysed the data independently and met during analysis to revise the coding frame. The frame was then further revised through discussions with the focus group facilitators. Service user data were coded separately from staff data to identify specific intra-group themes. Themes and sub-themes relating to the explanation of SLaM C4C were noted; these formed the basis of training materials to help clinicians explain the process to people on their caseload.

## Results

Staff focus groups included a mix of professions (social worker, clinical psychologist, community psychiatric nurse) and included both team members and team managers. Service user focus groups reflected the demographics of the Trust population. Sample demographics for service users and staff are shown in Table 1.

**Table 1:** **Participant demographic data**

**Group**	**Number**	**Gender**		**Ethnicity**				
		***M***	***F***	***Black***	***White British***	***White Irish***	***Indian***	***Mixed***
Service users	26	16	10	11	12	1	1	1
*Staff*	17	6	11	6	11	0	0	0
Total	**43**	**22**	**21**	**17**	**23**	**1**	**1**	**1**

Across staff and service user focus groups, themes broadly fell into two categories: firstly benefits about C4C and potential concerns around confidentiality and consent - and secondly recommendations for the way C4C could be implemented, including approaching service users, communicating clearly and allaying anxieties. Within these categories staff and service user responses varied.

### Staff focus groups

#### Benefits and potential concerns about C4C

Some staff reported that a C4C register would make it easier for people to participate in research, which would benefit both the service user and the researcher.

“I like the idea that we don’t have to be involved and that we’re not…nanny keepers […] that you don’t have to go through an anxious clinician or care coordinator. […] I do like the idea that people can make their own decisions a bit.” (Staff, Early Intervention, P4)

“It’s beneficial for the researcher as well as the patient, it flags up that they’re quite interested.” (Staff, Inpatient Unit, P1)

Others expressed concerns about explaining the process to service users, which would need to be addressed through safeguards. Firstly, staff argued that because C4C is consent for *future* contact, some service users might forget that they consented and this might undermine their relationship of trust with their care co-ordinator:

“I think you might feel quite uncomfortable in a situation whereby someone was being contacted by a researcher … and then all of a sudden a situation might arise where they’re saying, I don’t remember doing this and you were the care coordinator and now you’re in a conflict with your own client” (Staff, Community Care, P2)

Secondly, participants across all groups worried that they would find it difficult to justify why researchers could potentially have access to the entirety of their clients’ records. Some feared that service users might see this as a breach of confidentiality, which might pose a further challenge to the relationship between care co-ordinators and their clients:

“it’s really dodgy… it’s alright having access to your diagnosis and medication and whatnot, but I think when it gets that deep [i.e. researchers seeing clinicians’ detailed notes] I’m not sure I could justify why they would need access to that.” (Staff, Early Intervention, P2)

Thirdly, C4C was seen as an ‘abstract concept’, which might be difficult to explain to service users. Concerns of this nature were most keenly felt for staff in wards. Some staff feared that seeking C4C may trigger paranoid ideation in some service users. Clear presentation may allay this problem:

“whoever explains this to the patient would have to be absolutely crystal clear because when patients have paranoid delusions about people trying to get them or people trying to use information on them …it can seem quite broad […] the safeguards for it would have to be really specifically explained …otherwise I think loads of people will say no just on the basis that they don’t know what’s going to be done with their information.” (Staff, Inpatient Unit, P3)

Finally, staff in all three focus groups reported anxiety over the prospect of no longer participating in research recruitment. It was felt that care co-ordinators have a unique and informed access to patients which cannot easily be duplicated by researchers or other clinicians:

“I feel it’s good practice … that we as care coordinators will link up with the clients to explain situations to them before we can give out to you.” (Staff, Early Intervention, P3)

“…there is something useful about the care coordinator being tied into what’s going on. … they add value in terms of making it a safer, better way of collecting new information. So I guess my anxiety would be around how you’d ensure those safeguards are still in place” (Staff, Community Care, P5)

#### Approaching service users about C4C

There was agreement that service users should only be approached about C4C when they are stable. Teams concurred that, while the exact time should be left to the clinician’s discretion, the point of entry to services should be avoided, since this is often a moment of crisis:

“I think that’s a really, really difficult time to ask people presenting [to our intake service] because they’re in crisis of one sort or another so to me that would not be appropriate. And equally I think approaching people whilst they’re in hospital is quite difficult, for this kind of blanket thing.” (Staff, Community Care, P5)

Those in early intervention services made the additional point that service users who are not yet familiar with services may feel under pressure to consent, because they may assume that withholding consent will affect their care:

“I think a lot of clients can’t separate […] they feel they ought to [consent] because it’s presumably part of their care, not realising it’s nothing to do with their care at all … it can sometimes feel a bit unfair when people are quite vulnerable … to get them to agree to stuff” (Staff, Early Intervention, P2)

Participants argued that, since C4C might seem too much of an abstract concept for some people, care should be taken to present it in a clear and concrete fashion, for example by highlighting the benefits of research:

“I think … signing up to this sounds worse than asking people about individual research projects, a more anxiety-laden commitment. People might not want to sign up to this and therefore it excludes them from being contacted about any research” (Staff, Early Intervention, P2)

“I think it might be informative [to tell them]… this is the organisation you are part of and the philosophy is to be a centre of excellence and to do research that helps people in a clinical way and you could be part of this.” (Staff, Early Intervention, P4)

Equally, the consultation should take into account different literacy requirements:

“Some people have got learning difficulties, people don’t have great English, there’s a sense of how we help them to retain that information as well isn’t there, …something we can embed so when you guys do ring up they don’t just go, no I don’t want to buy your nonsense, put the phone down. I think it’s case by case again, really. I think you’d need to give us a range of differently pitched information that would be helpful.” (Staff, Community Care, P5)

Service users should be made aware that they can customise their entry on the C4C register. Some clinicians suggested that specific prompts should also be included, as those unfamiliar with research practice may not always be able to think of reasonable restrictions:

“Would you have prompts that, oh, you’d like to be contacted for this but not this? Because someone might not know” (Staff, Early Intervention, P1)

Furthermore, clinicians across all groups suggested that service users should be asked periodically as circumstances and mental capacity may fluctuate.

“Maybe you could review it yearly to check, maybe that’s not necessary if they can opt-out at any time, whether you wanted to have it updated to check the capacity is there still [happy].” (Staff, Early Intervention)

Finally, staff working in inpatient wards felt that a leaflet may further allay people’s anxieties by making research less abstract and thereby less threatening

“So the clinician sits down… with that leaflet and explains to the patient, gives them examples of some of the research ….it could help to clarify things for them.” (Staff, Inpatient Unit, P3)

### Service user focus groups

#### Benefits and potential concerns about C4C

Service users across all the focus groups felt that C4C streamlined research practice and, in particular, made it easier for researchers to match people to specific studies. Some reported that this system was an improvement over conventional recruitment methods because it enabled service users to choose the terms of their participation, rather than leaving this judgement to their care co-ordinator:

“I think that if it helps […] to get the right people to do the right research then I’m all for it.” (Service user, Research Negative, P4)

“Personally I think it’s a good idea because it allows people to be contacted directly, it gives their consent before anyone else can say what they should do […] You can’t customise your care coordinator, do you understand what I’m saying? I think this one’s alright, you can modify it to your needs and how you want it…” (Service user, Early Intervention, P2)

There were two overarching concerns about C4C: participants worried about breaches of confidentiality and about being forced to consent. Anxieties over possible leaks of confidential information were reported in three of the focus groups. These were motivated, at least in part, by some participants’ perception that their psychiatric records contained inaccurate and possibly stigmatising information:

“And when someone’s delusional while they’re under the influence of medicine, these things half the time mean nothing because you talk a load of drivel …. but it comes up in your notes and it goes to authorities that shouldn’t have these things. It’s not right and after a certain period of years some of this stuff should be deleted.” (Service user, Community Care, P1)

Some participants were uncomfortable with the idea that consenting for contact would allow researchers unknown to them to access their files. These participants imagined that joining such a research register may result in loss of confidentiality and indiscriminate sharing of records which they imagined to be inaccurate and therefore ‘dangerous’:

“… if I had consented to consent, I don’t know who is looking at my files, it might be people who I think are really disreputable or whatever [….] Files… can be very misleading, incorrect and even dangerous and I think the less they’re shared freely, as if they were gossip, the better.” (Service user, Community Care, P5)

Additionally, references to records being shared mobilised wider anxieties about government surveillance in at least one participant.

“this awful government we’ve got… I can imagine them doing anything they could do to get into people’s health records, to cut people’s healthcare, to make them work when they’re sick, to make their families pay for their healthcare when they’re NHS patients, all sorts of things could go wrong and what’s safeguards have you got for that?” (Service user, Inpatient Unit, P4)

Not being in control of participation was a shared concern. Some worried that they might consent when unwell or might forget that they gave consent:

“People can be vulnerable. […] if I’d been in hospital and somebody had asked me, I might’ve given consent, forgotten all about it, been contacted in a year’s time by six researchers and, you know, been exploited and all the rest.” (Service user, Early Intervention, P5)

Some participants wanted assurances that consenting for contact will not lead to forced participation in studies, and compared this to their experiences of involuntary treatment. These concerns were most marked amongst service users in the ‘research negative’ group, where some participants objected to the idea of being put on ‘a list’ or ‘a database’ as they associated this with being singled out and with being unable to exercise choice over participation.

“you could do [research] when you have time, when you feel it’s not forcing you to go and do it. Like how we are forced to take medication […] we don’t want to go on those [studies] … when we don’t have time for these studies, or when [we] are forced to come” (Service user, Black and Minority Ethnic group, P3)

“You don’t have a database of healthy people in the thing, why should you want to keep one of the vulnerable ones? And especially a lot of them they need money, they’ll be needing money so they’ll say yes to anything” (Service user, Research Negative, P2)

#### Approaching service users about C4C

While there was no overall agreement across all five groups, participants stressed the importance of having C4C presented by a trusted clinician. Some participants in the ‘research negative’ group pointed out that another option should be available, as some service users may not have a trusting relationship with their clinician.

“They [the doctors] are not the ones who’ve been meeting with you every week, every month, it’s your care coordinator. Obviously she knows you better, she knows what you’re like” (Service user, Early Intervention, P2)

“Some people don’t get on with their clinicians […] not only that, they’re not trusted so they don’t want them to know what they’re doing. Shouldn’t go through the social workers” (Service user, Research Negative, P6)

Participants concurred that a person who feels stable, is not in hospital and has become relatively familiar with services is in a better position to make an informed decision about research. At the same time, participants felt that service users’ needs are different, and therefore the decision to approach a service user about C4C should be made on a case-by-case basis:

“probably it should be asked …, [when you] know … what’s happening with your treatment and then you can probably decide if you want to take part … because for a lot of people starting a new service, it could be a little overwhelming” (Service user, Early Intervention, P3)

Most groups agreed that service users should be able to stipulate frequency and manner of contact in order to avoid being contacted too often. At the same time, participants worried that service users may not always be sufficiently knowledgeable about research to provide examples of when/how, and about what they might be contacted. They may benefit from prompting:

“And should there be a limit on how many times a person can be approached, you know, because, there’s maybe a lot of people researching and they keep hitting the same person because they turn up …” (Service user, Research Negative, P4)

“sometimes you sit there [and] you can’t think of anything whereas if they ask you specific questions, you can be able to say yes, no, maybe, this amount of times, that kind of thing.” (Service user, Early Intervention, P3)

Given the weight of concerns over confidentiality, participants wanted clarification about how their data would be accessed and how their privacy would be protected:

“We have to look at how [researchers] are policed first, [….] the numbers first of all. Restrict the access to the information and of course what is the reason for wanting permission?” (Service user, Community Care, P6)

Two participants in the ‘research negative’ group summarised well many of the concerns expressed among service users, with statements relating to the safeguards they would need in order to opt in to a C4C register:

“To have this explained to me in a language that I can understand. To recognise that I’m never ever going to be exploited, that I’m never going to be coerced into doing something that I’m not comfortable with and that there was a body of people that’s there to protect my rights.” (Service user, Research Negative, P5)

“to be able to opt-out immediately without any fuss.” (Service user, Research Negative, P4)

## Discussion

Our data show some convergence between staff and service user perceptions of SLaM C4C. We found that both staff and service users were positive about C4C and reported that an EHR-based research register is likely to expedite research and offer service users more control over participation. Additionally, they both suggested that the idea of researchers having access to clinical records was one of the most challenging aspects of the process. Furthermore, staff concerns that the ‘abstract’ nature of consenting for contact may generate misunderstandings or paranoid ideation appeared to be borne out by some participants in the service user groups.

Some differences of emphasis between focus groups were noted. For the most part, these differences were not marked, and resulted from individual participants expressing strong views. Two trends were apparent: staff participants from the inpatient ward were more likely to suggest that C4C may not be suitable for people who experience paranoid ideation. Equally, service user participants in the ‘research negative’ group were more likely to worry about being forced to consent, and were more concerned with ways of managing researchers’ access to records. There were no overall differences related to service user ethnicity or experience of services.

We need to consider, however, that the thematic convergence between staff and service user concerns may be more apparent than real, as staff may not always correctly anticipate service user concerns about some aspects of C4C. For example, staff suggested that service user anxieties over being pressured to participate in C4C could indicate paranoid ideation or a lack of understanding of what informed consent means (i.e. that it involves the exercise of choice). There is however, an alternative interpretation: these anxieties may relate to some service users’ association of mental health services with coercion and involuntary treatment, that is, with a *loss* of choice. It is possible that, for some, this association extends to mental health research. The initial C4C consultation would therefore need to navigate this concern by clearly distinguishing between research and treatment and by highlighting the opt-in nature of the register.

Equally, while service user and staff participants both wished to limit researchers’ ability to access clinical records, their motivations differed. Staff discomfort over researcher access to records was motivated by their concern that service users might see such access as a breach of trust between clinician and client. On the other hand, service users were concerned that their records may be inaccurate, and became anxious that access to records might compromise their dignity and welfare (as one participant put it, records may be shared as if ‘they were gossip’). Concerns over record validity are neither unwarranted nor unique to mental health: indeed, studies have shown that the digitalisation of health records may result in a proliferation of errors [13-15]. While service user anxieties over illegitimate sharing of records may suggest a lack of familiarity with research practice and governance, they may also stem from participants’ perception that records are stigmatising documents. Therefore, greater transparency around the content and construction of clinical records would be useful in implementing an EHR-linked research register. Studies indicate that better access to health records is more broadly beneficial because it encourages collaborative decision making and adherence to treatment [16,17]. Emerging initiatives in the introduction of patient generated records may prove an important step in this direction ^c^.

Once the main concerns of service users and clinicians were identified, a training protocol for C4C implementation was generated. The protocol specified that simple language should be used throughout and that the benefits of research should be highlighted through the use of examples. Clinicians were presented with a list of ‘Frequently Asked Questions’ which could be used as a template in their consultations with service users. The list:

- Emphasised the opt-in and voluntary nature of C4C
- Highlighted the safeguards around researchers’ access to records
- Clarified that participation in C4C does not bind service users to subsequent participation in specific research projects, and that the latter can be decided independently
- Specified service users’ right to change their decision at any time.

The training protocol thus addressed service user concerns around coercion and illegitimate access to records. While these concerns were more prominent in the ‘research negative’ group, their presence had been noted across service user and staff groups, thereby justifying their prioritisation in the protocol. The C4C consultation would be supplemented by an information leaflet which service users could take home to remind them of their decision.

Additionally, our findings suggest that a training protocol may not always be sufficient in addressing service user concerns and that the implementation of a novel framework for research participation may call for broader structural changes. In this respect, transparency around research practice, a broader Trust-wide re-conceptualization of research as the foundation for clinical treatment and services and principled service user involvement in research governance, may be a useful way forward.

### Limitations of the study

The study relates to the operationalisation of the ‘Consent for Contact’ model within an English mental health context and is consequently tailored to the provisions for health record confidentiality and research governance under UK law. Therefore, our findings may not be transferable to countries where the use of health data for research is not equally constrained by privacy legislation (e.g. Scandinavian countries) [18]. Furthermore, as our study was conducted in a large UK metropolitan mental health Trust, with a highly ethnically diverse population and high levels of social and economic deprivation, our findings may not be generalizable to non-metropolitan locations. Finally, since our study was conducted in psychosis services, our training protocol would need to be adjusted for use in other mental health services – for example, in services for people with substance misuse problems, where concerns about confidentiality may be more prevalent ^d^.

## Conclusions

Findings indicate that both staff and service users associate an EHR-linked register with greater service user autonomy and more favourable conditions for research recruitment. Acceptance among staff and service users is dependent upon presenting the information to allay anxieties around coercion, degree of commitment, and about how records are going to be used. Since clinicians will be introducing C4C to service users, they need to be trained in how best to present this information. For some service users, researcher access to records is likely to be the most challenging aspect of the consultation.

## Endnotes

^a^The NHS constitution states: *“the NHS will do all it can to ensure that patients, from every part of England, are made aware of research that is of particular relevance to them. The NHS is therefore putting in place procedures to ensure that patients are notified of opportunities to join in relevant ethically approved research and will be free to choose whether they wish to do so.” 3. Department of Health:****The NHS Constitution****. In. London: Crown; 2013.*

^*b*^For information on SLaM ‘consent for contact’ see http://www.slam.nhs.uk/research/patient-involvement/current-opportunities/consent-for-contact.

^c^One such resource is ‘My health locker’ launched in South London and Maudsley mental health NHS Trust in 2012. ‘My health locker’ is an online tool which enables service users to monitor their health, view parts of their record and care plan, provide feedback on their treatment and keep track of appointments (see http://www.slam.nhs.uk/patients-and-carers/patient-information/myhealthlocker).

^d^In the UK, addiction or substance misuse teams are normally part of mental health services.
